# A Time-Motion Analysis of Turns Performed by Highly Ranked Viennese Waltz Dancers

**DOI:** 10.2478/hukin-2013-0025

**Published:** 2013-07-05

**Authors:** Jerneja Prosen, Nic James, Lygeri Dimitriou, Janez Perš, Goran Vučković

**Affiliations:** 1 University of Ljubljana, Faculty of Sport, Slovenia.; 2 Middlesex University, School of Health and Education, London.; 3 University of Ljubljana, Faculty of Electrical Engineering, Slovenia.; 4 University of Ljubljana, Faculty of Sport, Slovenia.

**Keywords:** Dance Sport, Ballroom dance, movement, turns, speed

## Abstract

Twenty-four dance couples performing at the 2011 IDSF (International DanceSport Federation) International Slovenia Open were divided into two groups: the first twelve placed couples (top ranked) and the last twelve placed couples (lower ranked). Video recordings were processed automatically using computer vision tracking algorithms under operator supervision to calculate movement parameters. Time and speed of movement were analysed during single natural (right) and reverse (left) turns performed during the Viennese waltz. Both top and lower ranked dancers tended to perform similar proportionate frequencies of reverse (≈ 35%) and natural (≈ 65%) turns. Analysis of reverse turns showed that the top ranked dancers performed less turns on a curved trajectory (16%) than the lower ranked dancers (33%). The top ranked couples performed all turns at similar speeds (F = 1.31, df = 3, p = 0.27; mean = 2.09m/s) all of which were significantly quicker than the lower ranked couples (mean = 1.94m/s), the greatest differences found for reverse turns (12.43% faster for curved trajectories, 8.42% for straight trajectories). This suggests that the ability to maintain a high speed in the more difficult turns, particularly the reverse turns on a curved trajectory, results in the overall dance appearing more fluent as the speed of movement does not fluctuate as much. This aspect of performance needs to be improved by lower ranked dancers if they wish to improve rating of their performance. Future research should determine which factors relate to the speed of turns.

## Introduction

Competitive dance (DanceSport) consists of Latin-American and ballroom disciplines, both of which include five dances that contribute equally to the final score attributed to dance couples. In ballroom, the Waltz, Tango, Viennese waltz, Slow foxtrot and Quickstep are danced by couples who typically adopt a closed-hold position and perform set figures as well as their own moves using both creative footwork and stylish actions ( Pittman et al., 2005 ). To be a successful dancer, a couple must move as one, trying to maintain fluid movements in time with the music, regardless of changes in direction and planned pathways ( Kassing and Jay, 2003 ). This is undertaken in the presence of other dancers ( Tremayne and Ballinger, 2008 ) who occupy the dance floor at the same time and consequently trajectories cannot be completely planned in advance. Zaletel et al. (2010) found that each individual within a couple (three adult Internationals and three elite youths) travelled very similar distances with less than 1.21% difference between them.

The Viennese waltz is the classic original waltz involving continuous turns, where couples rotate around each other, danced at a tempo of 58–60 bars (180 beats) per minute. This is characterized by powerful rotational body movements on the first beat of each bar, by swaying toward the centre of turns, with couples rising high on the toes of both feet during the second and third beats of the music ( Laird, 1994 ). In terms of directional characteristics, the Viennese waltz always follows an anticlockwise trajectory with rotational movements around the path of a couple in either the natural turn (to the right) or the reverse turn (to the left). It is the way in which a dance couple choreographs these movements (figures, turns and trajectories) in relation to the music that determines success ( Donald, 2012 ).

The importance associated with the dancers’ movements can, to some extent, be determined by examining the judging criteria used to classify couples in rank order in competitions. The criteria used for assessment are timing, rhythm, technique, body movement, bodylines and expression, which are well defined but interrelated and therefore complex to objectively evaluate. The judges, or “adjudicators” as they are called in DanceSport, also have to evaluate the correctness of the technique. For example, body movement, body-swing and balance during dancing are defined by the World DanceSport Federation ( WDSF, 2012 )

Consequently the tactics in DanceSport relate to the choice of choreography for each dance such that this can best facilitate the appropriate movement patterns that both work well with the music and present the correct movements to the “adjudicators” ( Uznović et al., 2002 ).

Since the quality of a dancer’s performance depends on skilful production of motor skills within the constraints of the dance type it is suggested that a number of different skills can be influential on dance success ( Kostić et al., 2004 ; Lukić et al., 2011 ). Uznović et al. (2009) suggested that the speed of movement is important for success in the Viennese Waltz and the Quickstep. Similarly, Zaletel et al. (2010) found that on average International adult dancers were 0.3 m/s faster than elite youth couples during the Viennese waltz. However the speed of movement in this study was presented as a mean value for the duration of a single dance and was calculated on only three dance couples. A more detailed analysis of dancers’ individual movements was not carried out. The choreography in Viennese waltz is relatively simple, consisting mainly of turns, thus a more detailed time-motion analysis of dancers’ choreography, in particular the turns, would be appropriate. A turn in dance denotes external rotation of the lower extremities as a result of interdependent hip, knee, lower-leg and the foot-ankle complex movements ( Champion, 2008 ). Dancers must have adequate length and strength in the structures around the trunk, the hip joint and the ankle joint ( Grossman, 2005 ) to make the perfect turn which requires 90° of external rotation of each leg. Most investigators agree that approximately 60–70° of the turn is generated at the hip joint and only 10–35° from the knee and below. The focus of this research was a comparison between natural and reverse turns performed by top and lower ranked couples ( Champion, 2008 ; Welsh, 2008 ).

The number of natural and reverse turns were analysed to determine whether speed of movement within a single turn differed between the top (n=12) and lowest ranked (n=12) couples performing the Viennese waltz at an International competition.

## Material and Methods

### Participants

Twenty-four couples, performing in the adult (over 19) category of the 2011 IDSF International Slovenia Open dance competiton were selected for analysis as they finished in the top and bottom 12 of the 48 couples in the competition. Mean age and body height of female dancers was 22±2.9 years and 167±4.5cm and of male dancers 24±2.4 years and 178±4.9cm, respectively. The mean years of dance experience per couple was 13±2.9 years. No significant differences (t-tests) were found for age, body height or dance experience between the top and lower ranked dancers (p > 0.05). The top 12 dancers were filmed dancing in the final or semi-final stages where there were six couples dancing together. The bottom 12 did not reach the quarter-final stage and were filmed dancing during the preliminary stages where twelve couples danced together. The lower ranked couples danced for a slightly longer duration (mean = 75.26s dancing time) in comparison to the top ranked couples (mean = 67.24s) because competition rules dictate that more time is allocated when more dancers are competing together as this adjustment facilitates the judging process.

Ethical approval was granted by a University committee and informed consent was obtained from all participants.

### Procedures

**Figure 1:**
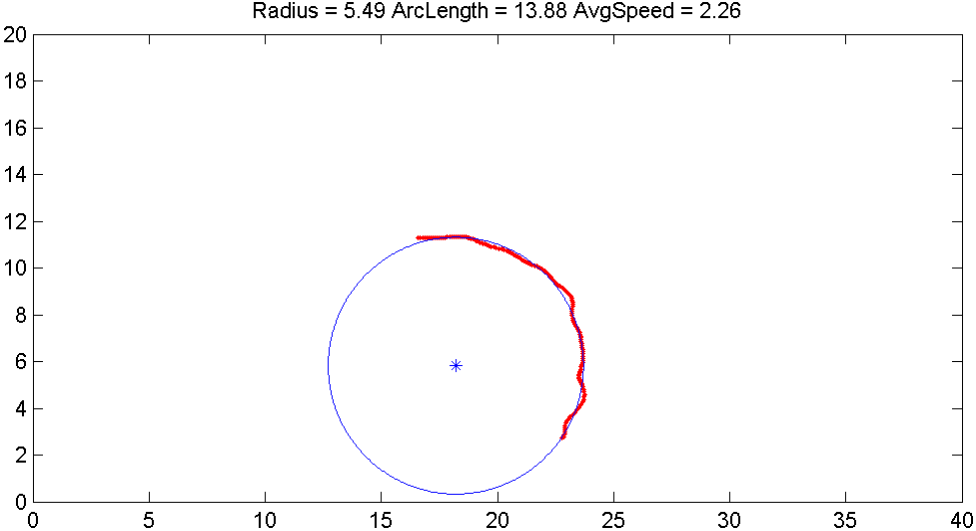
*
The output of the circle fitting method on a curved section of the trajectory
*

All dances were recorded using a fixed analog PAL video camera (JBL UTC – A6000H, Korea), fastened to the ceiling in the centre of the dance floor, its wide-angled lens (2.3 mm – 6.0 mm, Kenko, Japan) were adjusted to cover the entire dance floor which was 26 x 15 m. The video signal was recorded directly to DVD+R disc, using a Phillips DVD recorder, yielding a digital MPEG2 encoded file that was transferred to a personal computer. Before further processing the video was de-interlaced and resampled to a resolution of 352x288 pixels and frame rate of 25 frames per second. Spatial calibration was performed to provide plane-to-plane mapping of image pixel locations into the world coordinate system of the dance floor. This video was processed automatically using state-of-the-art computer vision tracking algorithms ( Kristan, 2009 ) under the supervision of the operator, who was responsible for detecting and correcting any mistakes made by the automated tracker. Every couple was tracked from the beginning until the end of their Viennese waltz. Output from the tracking software was further processed, as detailed by Perš et al. (2002) , to yield speed and path length information. Additionally, operators manually annotated curved sections of the trajectory. For each of the curved sections, an algebraic circle fit method ( Taubin, 1991 ) was used to derive the arc radius and the arc length. The implementation of the method in Matlab language is publicly available: http://people.cas.uab.edu/~mosya/cl/MATLABcircle.html. An example result of a circle fit on a curved section of the trajectory is shown in Figure 1 . (e.g. insert Figure 1 here)

A second camera was located by the side of the dance floor to facilitate annotation of the details of the dancers’ posture relationships. These digital images were transferred into the same computer and temporally synchronized with the video from the top-view camera. The operator manually annotated these images. All processed data were stored using Microsoft Access software and filtered using SQL queries.

### Statistical Analysis

**Figure 2:**
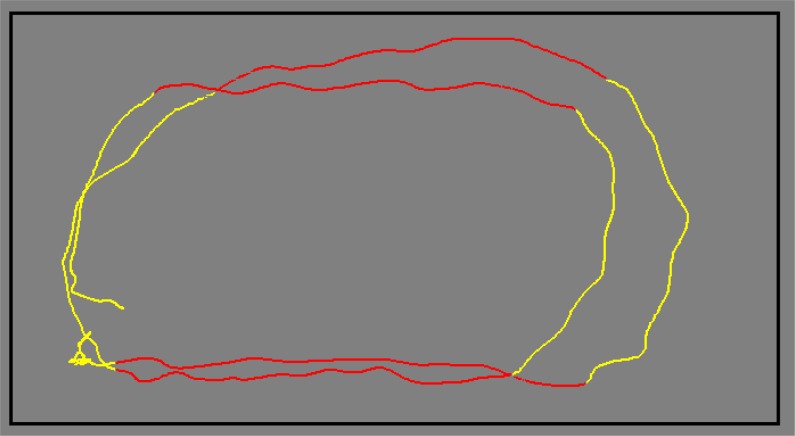
*
Movement on a straight (red line) and curved (yellow line) trajectory
*

Data analyses were undertaken in the SPSS statistical package (v 17.0) with the data separated into two groups (the top and lower 12 couples). Dependent variables were the total time and average speed of movement (m/s) for each single full turn (the dance couple rotates through 360 degrees). A single full turn was deemed to have started when one of the dancer’s feet left the ground, just before the first step of the turn and finished after six steps when both feet were on the ground. These turns can be executed in either clockwise (referred to in dance as a natural turn, NT) or anti-clockwise (known as a reverse turn, RT) directions. Dancers also move around the dance floor in an anti-clockwise direction, either on a curved or straight trajectory ( Figure 2 ), and so turns were also notated in terms of the overall trajectory in which the dance was moving. (e.g. insert Figure 2 here)

Hence, turns could be natural turns on a straight trajectory (NS), natural turns on a curved trajectory (NC), reverse turns on a straight trajectory (RS) and reverse turns on a curved trajectory (RC). The frequency of each turn type was compared between the top and lower ranked couples using the chi square test for independence and effect size determined using Cramer’s phi. Descriptive analyses were carried out including the Levene’s test for equality of variances and subsequently independent samples tests were used to determine differences between the top and lower ranked couples and between natural and reverse turns within each group. Two way ANOVAs with post hoc Scheffé tests were used to determine within (different turns) and between group differences for turn speeds, arc radii and arc lengths. Statistical significance was accepted at p < 0.05.

## Results

Very small correlations were found between the speed of dancers during turns and the age, body height or dance experience for either top or lower ranked dancers (correlation coefficients between −0.20 and 0.08). When the radii of the turns were compared there was no significant interaction (F = 0.46, df = 3, 66, p = 0.71, partial eta2 = 0.02) and no main effect for dancing standard (F = 0.03, df = 1, 22, p = 0.87, partial eta2 = 0.0) or turn (F = 0.57, df = 3, 66, p = 0.64, partial eta2 = 0.03). Similarly when the arc lengths were compared, there was no significant interaction (F = 0.34, df = 3, 66, p = 0.80, partial eta2 = 0.02) and no main effect for dancing standard (F = 0.24, df = 1, 22, p = 0.63, partial eta2 = 0.01) or turn (F = 1.09, df = 3, 66, p = 0.36, partial eta2 = 0.05).

Of the total turns the top ranked dancers performed 42.7% NC and 22.1% NS which was similar to the low ranked dancers (39.6% NC and 24.2% NS). However for reverse turns the top ranked dancers performed 5.7% RC and 29.4% RS which was different (chi-square = 8.199, df = 3, p < 0.05, phi = 0.12) to the low ranked dancers (12.1% RC and 24.2% RS). Thus, the top ranked dancers performed less of their reverse turns on a curved trajectory (16%; i.e. 5.7/(5.7+29.4)) than straight (84%), which was a lower proportion than the lower ranked dancers (33%; i.e. 12.1/(12.1+24.2)).

No differences were found in the duration of natural turns or reverse turns on a straight trajectory between the top and lower ranked couples. However, the top ranked couples performed reverse turns on a curved trajectory significantly slower than the lower ranked couples ( Table 1 ). (e.g. insert Table 1 here)

The top ranked couples performed the turns significantly quicker than the lower ranked couples (F = 7.30, df = 1, 22, p < 0.05, partial eta2 = 0.25). However, it appeared that the greatest differences were for reverse turns (12.43% faster for curved trajectory, 8.42% straight) compared to natural turns (7.04% faster for curved trajectory, 6.74% straight; Table 2 ). (e.g. insert Table 2 here)

When within couple differences were examined it was clear that the top ranked couples performed reverse and natural turns at similar speeds for both straight (t = 0.170, df = 133, p = 0.866) and curved trajectories (t = 0.675, df = 125, p = 0.501). Similarly the lower ranked couples also performed reverse and natural turns at similar speeds when on a straight trajectory (t = 0.677, df = 142, p = 0.499) but were significantly slower on reverse turns (mean = 1.85m/s) in comparison to natural turns (mean = 1.99m/s) when on a curved trajectory (t = 3.077, df = 152, p < 0.01; Table 2 ). One-way ANOVAs showed that the top ranked dancers performed all of their turns at similar speeds (F = 1.31, df = 3, p = 0.27) whereas the lower ranked dancers performed their turns at different speeds (F = 3.95, df = 3, p < 0.01). Post hoc Scheffé tests showed that the low ranked dancers performed the NC quicker than the RC (p < 0.05).

## Discussion

Dances are performed in time to the same tempo of music in the Viennese waltz suggesting that movement speeds would be similar between couples. This study analysed the quantity and speed of turns to determine whether there were differences between top and lower ranked couples. It was noted that the number of dancers on the dance floor dictated the total duration of a dance. This contributed to the finding that the lower ranked dancers performed more turns than the top ranked dancers even though there was no significant differences in duration of NS, NC and RS turns. Whilst both top and lower ranked dancers tended to perform similar proportionate frequencies of reverse (≈35%) and natural (≈ 65%) turns the top ranked dancers performed less reverse turns on a curved trajectory (16%) than the lower ranked dancers (33%). This suggests that top level dancers choreograph their routine in a way that reduces the frequency of the reverse turns on a curved trajectory, whereas lower level dancers were less able to do this. The reverse turn is more difficult to perform, particularly when on a curved trajectory, because the reverse turn tends to take the dancers in the opposite direction to the overall dance trajectory. Howard (2002) explains that when a reverse turn on a curved trajectory is performed the distance covered is 1/8 greater compared to the same turn on a straight trajectory, and when a natural turn on a curved trajectory is performed the distance covered is 1/8 less. Grossman et al. (2008) found that tibial torsion of the right leg was on average 2° greater than for the left leg, which may influence the difficulty of the reverse turn performance. A further explanation as to why performing turns on curved trajectories is more difficult than on straight trajectories is that the partner on the inside position needs to take smaller steps in comparison to the partner on the outside, with this relationship changing throughout the turn.

The top ranked dancers performed their turns with a higher speed (mean = 2.09m/s) than the lower ranked dancers (mean = 1.94m/s) and performed the different turns at similar speeds. In comparison, the lower ranked dancers were unable to maintain the same speed for the reverse turn on a curved trajectory in comparison to the natural turn on a curved trajectory. Since the arc radii and arc lengths of the turns were not significantly different between the top and lower ranked couples, this suggests that only the highest skill level dancers can perform the reverse turn at the same speed as the other turns. Since the lower ranked couples were unable to perform the reverse turns, particularly when on a curved trajectory, at the same speed as the natural turns, their speed of movement tended to rise and fall. This is likely to have contributed to a less flowing style of movement for the dance as a whole and visually does not create the softness of movement that the “adjudicators” look for. The most likely explanation for the lower ranked couples having lower speed during reverse turns is that their technique was flawed to some extent. Research suggests that dancers can have inaccurate perceptions of the joint action and muscle use during turns, be uncertain of the amount of turnout they have or how to use it properly ( Grossman, 2005 ; Daniels, 2007 ). It is thus important for dancers to realize that improving the actions of the hip, pelvis and spine can produce longer leaps, cleaner turns and hence more effective turns. The values for the lower ranked dancers are similar to the findings of a pilot study by Zaletel et al. (2010) who found that three national standard adult dancing couples averaged speeds of 1.89m/s when dancing in a non-competitive dance condition. It seems that the speed of movement in Viennese waltz increases in relation to the expertise of the dancing couple. This conjecture is supported in a study by Zaletel et al. (2010) who found that youth couples moved slower (1.62 m/s) than adult couples (1.89 m/s) but this paper measured all movements rather than just the turns.

In this study the age, body height and experience of the dancers were similar for the top and lower ranked couples and did not seem to have much influence on the speed of movement during turns. This speed probably depended more on other factors like technique and physical conditioning. For example, Grossman (2005) highlighted the importance of lower extremity muscles for producing good technique during turns as they can determine the ability to turn the leg outwards and hence affect the alignment during a turn. To improve a dancer’s performance, particularly the quality of turns, specific conditioning is required to optimize an individual’s range of movement such that the complex movement patterns through space can be achieved. By improving this ability couples would be able to maintain better technique and increase the speed of movement during both natural and reverse turns. This research suggests that lower speed of movement during turns in Viennese waltz can be an indicator of less than optimal physical conditioning which results in slightly compromised technique.

## Conclusion

It can be concluded that only very highly skilled dance couples are able to maintain the same speed for all turns, which are of differing difficulty due to the direction of the turn and the dance trajectory. The ability to maintain a high speed in the reverse turn on a curved trajectory leads to the overall dance appearing more fluent as the speed of movement does not fluctuate. Lower ranked couples seem to have difficulty in performing all turns at the same speed with the reverse turn on a curved trajectory the slowest in comparison to the other turns. From a coaching perspective this is the turn that should be practiced more often by lower ranked couples. However, this research does not identify why lower ranked couples find this particular turn more difficult although physical conditioning has been shown to affect technique and hence potentially compromise performance. Future research is required to determine whether problems tend to arise in terms of foot technique, the hold, bodylines or physical conditioning and how these parameters relate to the speed of movement and ultimately the quality of the dance.

## Figures and Tables

**Table 1:** *
Descriptive statistics and t-test results for the time of single full turns between the top and lower ranked couples.
*

Turns	Couples	N	Mean (s)	SD	df	t	Sig.
Natural turns straight (NTS)	Top	58	2.03	0.14	128	0.622	0.535
	Lower	72	2.05	0.17			
Natural turns curved (NTC)	Top	112	2.03	0.14	228	0.614	0.540
	Lower	118	2.01	0.18			
Reverse turns straight (RTS)	Top	77	2.02	0.14	147	0.941	0.348
	Lower	72	2.00	0.14			
Reverse turns curved (RTC)	Top	15	2.14	0.23	49	2.193	<0.05
	Lower	36	2.05	0.09			

**Table 2:** *
Descriptive statistics and t-test results for mean speed of single full turns between the top and lower ranked couples* .

Turns	Couples	N	Mean (m/s)	SD	df	t	Sig.
Natural turns	Top	58	2.06	0.31	128	2.445	<0.05
straight (NTS)	Lower	72	1.93	0.27			
Natural turns	Top	112	2.13	0.29	228	3.763	<0.001
curved (NTC)	Lower	118	1.99	0.26			
Reverse turns	Top	77	2.07	0.24	147	4.107	<0.001
straight (RTS)	Lower	72	1.90	0.24			
Reverse turns	Top	15	2.08	0.19	49	3.586	<0.01
curved (RTC)	Lower	36	1.85	0.21			
